# Interpreting and operationalizing the incurability requirement in Canada’s assisted dying legislation

**DOI:** 10.3389/fpsyt.2025.1549289

**Published:** 2025-03-17

**Authors:** Mona Gupta, Jocelyn Downie

**Affiliations:** ^1^ Départment de Psychiatrie et d’Addictologie, l’Université de Montréal, Montréal, QC, Canada; ^2^ Faculties of Law and Medicine, Dalhousie University, Halifax, NS, Canada

**Keywords:** assisted dying, incurability, Canada, eligibility, clinical assessment, law, medical assistance in dying

## Abstract

To access medical assistance in dying (MAiD) in Canada, a person must have a “grievous and irremediable medical condition” defined in part as “a serious and incurable illness, disease, or disability”. Thus, the clinical assessment of the incurability of a person’s condition is central to determining MAiD eligibility. However, the clinical interpretation and operationalization of the term have been uncertain due to the absence of a clear legal definition and evolving legislation. This has led to confusion and controversy in the public and professional discussion of MAiD eligibility. In this paper, we examine various attempts to interpret and operationalize the term “incurable”, identifying the limitations of each approach. We aim to overcome these limitations by proposing a method for operationalizing the term. We argue that our approach: (1) is consistent with the current legal framework, (2) is consistent with the interpretations of the terminology used in the *Criminal Code*, and (3) reflects the clinical knowledge and reasoning about the full range of medical conditions that can lead to a request for MAiD. In our analysis, we show that incurability cannot be understood only as a feature of a person’s medical condition but resides in the interplay between the nature of the pathology and the person’s treatment decision-making. Our analysis should help with the ongoing operationalization of the incurability requirement in Canada. It may also be helpful to clinicians in other jurisdictions that either invoke or are considering invoking similar terms/concepts.

## 1 Introduction

In February 2015, the Supreme Court of Canada, in *Carter v. Canada* ^1^, struck down the prohibition on medical assistance in dying (MAiD) found in the *Criminal Code of Canada*. In response, the federal Parliament passed Bill C-14, *An Act to amend the Criminal Code and to make related amendments to other Acts (medical assistance in dying)* ^2^, establishing that, in order to be eligible for MAiD, a person must have a “grievous and irremediable medical condition”, which was defined by four subelements: (1) “a serious and incurable illness, disease, or disability”; (2) “an advanced state of irreversible decline in capability”; (3) “enduring physical or psychological suffering that is intolerable to them and that cannot be relieved under conditions that they consider acceptable; and (4) “natural death has become reasonably foreseeable.”^3^

None of the key clinical terms within these four subelements were defined in the law, nor did the Federal Government offer any definitions. This initiated a multiyear process in which various groups attempted to interpret (explain the meaning of) and operationalize (put into use) these terms. In this paper, we focus on the term “incurable”.

There have been four inflection points in this process. First, when incurable was introduced into the law through Bill C-14 following the *Carter* case. Second, when “natural death has become reasonably foreseeable” was removed from the law through the *Truchon* decision^4^ and Bill C-7 (the federal Parliament’s response to the *Truchon* decision).^5^ Third, when Parliament introduced a new temporary exclusion from eligibility for persons with mental illness as their sole underlying medical condition (MI-SUMC) through Bill C-7 (until 2023).^6^ Fourth, when Parliament twice debated whether to extend the temporary exclusion (through Bill C-39 (extension until 2024) and Bill C-62 (extension until 2027).^7^ At each point, questions surfaced about the incurability criterion, particularly its relationship with the person’s treatment history and future treatment decisions.

For example:

- Can a person be said to have an incurable condition if they refuse treatment that might cure their condition (and where without it they will die)?
- Can a person whose natural death has not yet become reasonably foreseeable be said to meet the incurability criterion if they have refused some or many treatments for that condition?
- Can a mental illness ever be said to be incurable? If so, under what circumstances?

In this paper, we review the evolution of the operationalization of the incurability requirement for MAiD in Canada against the backdrop of these inflection points, which brought to attention different clinical conditions to which the term incurable had to be applied. First, we trace the inclusion of the incurability requirement in Canada’s federal MAiD law. We then engage with three primary sources that attempt to operationalize incurability. The first is an expert report published by the Institute for Research in Public Policy in 2019. We show how subsequent judicial (2019) and legislative (2021) developments posed challenges to the IRPP analysis. The second and third sources we explore are the reports of the two expert groups tasked with, among other things, analyzing the term—the Expert Panel on MAiD and Mental Illness (2022) and the Task Group on MAiD Model Practice Standards Task Group (2023). Drawing on the analyses laid out in these primary sources and their limitations (1–4), we offer a concrete, specific, and clinically grounded approach to operationalizing the incurability requirement in MAiD assessments within today’s legal context, as well as the context of MI-SUMC, which is set to become legal in 2027. We refined our proposal by testing it iteratively against relevant legislation, court decisions, and clinical concepts.

It is important to note at the outset that the term incurable can be mistakenly treated as synonymous with MAiD eligibility. This paper focuses on incurability due to the questions it has generated; however, this term is only one element of a grievous and irremediable medical condition, which itself is just one of several eligibility requirements under Canadian law. A person may well have an incurable condition, but this does not necessarily mean they qualify for MAiD. Therefore, in this paper, we propose a pathway to operationalizing incurability for clinical assessment purposes, not for determining MAiD eligibility as a whole.

This paper focuses on Canadian law and its use of the term “incurable”. Other countries that also allow assisted dying outside the end of life have similar requirements (5). For example, Dutch law states that the physician must: “be satisfied that the patient’s suffering is unbearable, with no prospect of improvement… have come to the conclusion, together with the patient, that there is no reasonable alternative in the patient’s situation”. Similarly, Belgian law requires that the physician must ensure that “the patient is in a medically futile condition of constant and unbearable physical or mental suffering that cannot be alleviated, resulting from a serious and incurable disorder caused by illness or accident” (6). Even jurisdictions that restrict assisted dying to patients with terminal illnesses indirectly invoke the concept of incurability, either within their definitions of “terminal disease” or directly in their eligibility criteria. For example, in Oregon, “[t]erminal disease” means an incurable and irreversible disease that has been medically confirmed and will, within reasonable medical judgment, produce death within six months.” In Victoria, Australia: “the person must be diagnosed with a disease, illness or medical condition that—is incurable”. Some permissive jurisdictions, such as Western Australia, have opted not to require incurability, arguing that it is unnecessary given other criteria—such as “advanced, progressive and will cause death”—and that it could impose autonomy-violating requirements regarding undergoing treatments (7, 8).

Despite differences in legislation, these jurisdictions have all had to (or will have to) address questions regarding the operationalization of these requirements (7, 8). Their approaches have informed our own while considering Canada’s specific legal, policy, and practice contexts. In addition to contributing to discussions in other jurisdictions, the analysis presented here may also help inform international debates, as several other jurisdictions are considering allowing some form of assisted dying.

## 2 Carter v. Canada

In *Carter v. Canada*, the Supreme Court of Canada (SCC) held that the existing *Criminal Code* prohibition of MAiD breached the *Canadian Charter of Rights and Freedoms*. The Court found that the prohibition violated the right to life, liberty, and security of the person (s.7) and the right to equality (s.15) and was not “demonstrably justified in a free and democratic society” (s.1 standard that must be met if limits on rights are to be allowed to stand by Canadian courts). ^8^ The SCC declared that the Criminal Code prohibition of MAiD was invalid insofar as it barred access to MAiD for individuals with “a grievous and irremediable medical condition (including an illness, disease or disability) that causes enduring suffering that is intolerable to the individual in the circumstances of his or her condition”. The Court further clarified that “irremediable” “does not require a patient to undertake treatments that are not acceptable to the individual”.^9^

While the SCC itself did not use the term “incurable”, its interpretation of the broader term “irremediable” provides context for the incurability requirement later introduced in Canadian MAiD law through Bill C-14.

## 3 Bill C-14

In response to the *Carter* decision, the federal Parliament passed Bill C-14, amending the *Criminal Code* and establishing the legal framework for MAiD in Canada. While the *Carter* decision stated that in order to be eligible for MAiD a person must have a “grievous and irremediable medical condition”, Bill C-14 further defined this expression by way of four subelements: “a serious and incurable illness, disease or disability”; “an advanced state of irreversible decline in capability”; “enduring physical or psychological suffering that is intolerable to them and that cannot be relieved under conditions that they consider acceptable”; and that “natural death has become reasonably foreseeable.” Bill C-14 introduced the term “incurable” as part of the MAiD eligibility requirements but did not define it.

Having no authoritative definition of the legislative provision, clinicians had no choice but to operationalize the term incurable themselves in order to assess eligibility for persons requesting MAiD. This posed a particular challenge because incurable is at once a word with a common dictionary definition, a term used in clinical parlance but without a single or uniform definition, and an undefined legal term used in the *Criminal Code* MAiD provisions with significant potential legal consequences for clinicians, including years of imprisonment.

For individuals who were already in a terminal phase of a certainly fatal condition, the operationalization of incurable seemed straightforward by virtue of the fact that death was both certain and proximate regardless of any clinical intervention. However, some individuals requesting MAiD were earlier in the disease trajectory, where a cure was possible or even probable with treatment. Could they be said to have an incurable condition if they were refusing the very treatment that might lead to a cure? In other words, is incurability a feature of the condition itself, the result of a person’s treatment decision-making, or some combination of the two? The process of operationalizing incurability required further effort.

## 4 IRPP Report 2018

In the absence of a statutory definition or judicial interpretation of incurable, in March 2018, the Institute for Research on Public Policy published “Interpreting Canada’s Medical Assistance in Dying Legislation” (IRPP Report) (1). This report proposed interpretations of keywords and phrases in Canada’s MAiD legislation developed through a Chatham House Rule^10^ process of engagement with an interdisciplinary group of experts.

The Report tried to articulate an interpretation of the terms (including incurable) that best respects established principles of statutory interpretation (9). These include, for example: context, purpose of the legislation, intent of Parliament, ordinary language, and technical meaning. The Report also took as its reference point legal considerations concerning refusals of potentially life-sustaining treatment, which was the most relevant analogous clinical context to MAiD requests, where natural death was reasonably foreseeable given that a person’s death was at stake in both situations. Canadian law has clearly established that capable individuals have the right to refuse any and all treatment even where the consequence of the refusal would certainly or might be death and even if the disease might be eliminated and/or their symptoms relieved had they accepted the treatment (10). The Report’s analysis also recognized the SCC comments about irremdiability quoted above.

It must be emphasized that the Report’s approach was developed when reasonably foreseeable natural death was an eligibility criterion, limiting access to assisted dying to conditions meeting this criterion. Clinically speaking, if a condition makes natural death foreseeable, its underlying pathophysiology must be sufficiently well understood to support that prediction. This allows clinicians to assess, probabilistically: (1) whether treatment can reverse or halt (cure) the condition’s pathophysiological process and (2) what is likely to happen without treatment.

In this context, the Report proposed the following interpretation of incurable and provided justifications for it:

“Incurable” means that, in the professional opinion of the medical or nurse practitioner, the person cannot be cured by means acceptable to that person. This does not mean that the professional opinion substitutes for the person’s assessment of whether the means are acceptable; rather it means that professional opinion holds that there are no clinical options that would accord with the person’s own assessment of acceptable means. (1 p.17)

The Report also emphasized that both the assessor and provider and the patient “have a role to play with respect to whether this eligibility criterion is met”.

The medical or nurse practitioner determines whether the patient has a condition and whether it can be cured by means acceptable to the person; the person requesting MAiD determines whether any potential treatments are acceptable.

Second, the established norms of informed consent apply in the context of MAiD; the provider must ensure that the patient has been informed of the treatment options and is capable of understanding and appreciating the consequences of refusing potentially effective treatment.

Third, good clinical practice also requires that the provider investigate a patient’s reasons for refusal of treatment to verify whether any of the concerns leading to rejection of a possible treatment may be mitigated. (1 p.19)

This interpretation was taken to mean the following in practice:

- The assessor determines whether there are any means available that might cure the patient’s condition.
- The assessor informs the patient requesting MAiD about any means that might cure their condition.
- If the patient refuses the treatment, the assessor explores the patient’s reasons for refusing and whether there are any means for mitigating those reasons.
- The assessor informs the patient requesting MAiD about any means that might mitigate their reasons for refusing.
- If the patient nonetheless refuses any means of curing their condition and mitigating any reasons they may have for refusing, and if the assessor determines that the patient’s condition will not be cured by the means acceptable to the person, then the assessor can form the opinion that the person’s condition is incurable.

On this approach, a person with, for example, bowel cancer with a 50% remission at 5 years with treatment could make an informed refusal of treatment and request MAiD. Given what is known about the disease trajectory without treatment, this informed refusal of potentially curative treatment could render the cancer incurable for the purpose of accessing MAiD. (But once again, it should be noted that even if this cancer is considered incurable, the person is not necessarily eligible to receive MAiD, as this is not the only eligibility requirement.) On the one hand, this scenario is difficult to reconcile with the standard clinical understanding of the term incurability as referring to the stage and severity of the medical condition itself, not a function of a person’s decisions. On the other hand, this scenario is compatible with the care options offered to someone who refuses a life-sustaining or lifesaving treatment; that is, the refusal of treatment does not render the person ineligible to receive other care options, such as palliative care, palliative sedation, or end-of-life measures, whenever they choose to receive them. This proposal was soon tested by further legislative developments.

## 5 Truchon v. Canada and Bill C-7

The next inflection point arose in *Truchon v. Canada*, where a Québec Superior Court Justice faced a *Charter* challenge to the *Criminal Code* provision requiring that a person’s natural death be reasonably foreseeable for MAiD eligibility.^11^ Justice Baudouin ruled that this provision violated the right to life, liberty, and security of the person (s. 7) and the right to equality (s. 15). She further determined that it was not demonstrably justified in a free and democratic society (s.1) and struck it down.

Since the incurability of the plaintiffs’ conditions was not in question (Jean Truchon had cerebral palsy and Nicole Gladu had postpolio syndrome), Justice Baudouin’s decision did not advance the legal understanding of the interpretation of incurable.^12^ However, *Truchon* significantly impacted the term’s operationalization by expanding the range of medical conditions eligible for MAiD.

In response to the *Truchon* decision, the federal Parliament passed Bill C-7, which removed “reasonably foreseeable natural death” from the MAiD eligibility criteria. It introduced two different sets of procedural safeguards to be followed depending on whether the requesters’ natural deaths were reasonably foreseeable (Track 1) or not (Track 2). Additionally, Bill C-7 specified that “mental illness” is not considered a “serious and incurable illness, disease, or disability” for MAiD eligibility. These changes had two major consequences.

First, the removal of “natural death has become reasonably foreseeable” expanded eligibility to a broader range of medical conditions. For some conditions, the underlying pathophysiology is well understood, allowing clinicians to determine that the disease process and its symptoms will not resolve or be relieved, even if the patient is not necessarily dying (e.g., congestive heart failure). In other cases, while the pathophysiology is understood, its long-term progression and symptom trajectory remain uncertain (e.g., multiple sclerosis). For yet other conditions, both the pathophysiology and its symptom evolution are poorly understood (e.g., complex regional pain syndrome). This broader range of potential medical conditions among MAiD applicants raised concerns about whether the previous operationalization of incurable—which relied on understanding the pathophysiology, disease progression, and trajectory toward death—remained inadequate. If a condition’s progression is uncertain (either the pathophysiology or the symptoms it causes), can it still be deemed incurable? Moreover, how does incurability apply if a patient refuses some or even many treatments that could alleviate their symptoms?

Second, the temporary exclusion of MI-SUMC highlighted questions about the incurability of mental illness—namely, whether a mental illness can ever be said to be incurable (11, 12), and if so, under what circumstances? Parliament effectively answered the first question by logical implication: if mental illness could never be incurable, the exclusion would be unnecessary—just as there is no need for exclusion clauses for universally understood curable conditions (for example, the common cold). Furthermore, in explaining/defending the temporary exclusion and its extensions, the Government acknowledged that mental illnesses could be irremediable (incurable illness, disease, or disability is part of the definition of an irremediable medical condition).^13^ Finally, it should also be noted that the Alberta Court of Appeal had previously determined that MI-SUMC could meet the *Carter* criteria for “grievous and irremediable medical condition”^14^, and the *Truchon* decision affirmed that *Carter* did not exclude MI-SUMC.^15^ In other words, Parliament and the Government’s position in Bill C-7 (and subsequent legislative discussions of the temporary exclusion) aligns with legal precedent.^16^ Thus, the first question set out above was answered.^17^ Mental illnesses can be incurable. The question remained, however, under what circumstances?

Attention therefore shifted to assessing the incurability of mental illness and determining how refusals of treatment should be considered in such evaluations. Anticipating the need for further reflection on the term incurable (among others) for individuals with mental illnesses, Bill C-7 mandated the Ministers of Justice and Health to commission an independent expert review “respecting recommended protocols, guidance and safeguards to apply to requests made for medical assistance in dying by persons who have a mental illness.”

## 6 Federal Expert Panel on MAiD and Mental Illness

The Federal Expert Panel on MAiD and Mental Illness published its report in May 2022 (2). It explored the consequences of removing the “natural death has become reasonably foreseeable” eligibility criterion, particularly for individuals whose sole underlying medical condition is a mental disorder.^18^

The Expert Panel noted that, in clinical parlance, the term incurable refers to the ability to make prognostic assessments based on knowledge of disease pathophysiology. However, since such knowledge does not exist for mental disorders, clinicians typically do not use this term when discussing them. As a result, the Panel made the following recommendation regarding the eventual operationalizing of the statutory provision that includes this term:

MAiD assessors should establish incurability with reference to treatment attempts made up to that point, outcomes of those treatments, and severity and duration of illness, disease, or disability.

It is not possible to provide fixed rules for how many treatment attempts, how many kinds of treatments, and over what period of time as this will vary according to the nature and severity of medical conditions the person has and their overall health status. This must be assessed on a case-by-case basis.

The Panel is of the view that the requester and assessors must come to a shared understanding that the person has a serious and incurable illness, disease, or disability. As with many chronic conditions, the incurability of a mental disorder cannot be established in the absence of multiple attempts at interventions with therapeutic aims. (2 p.11-12)

This approach indicated that incurability was both a characteristic of the condition (being refractory to treatments) and a result of a person’s decision-making (accepting many but not all treatments). The text implied that a patient might be considered to have an incurable condition if they have tried certain kinds of treatments, enough treatments, and over a sufficiently long period of time. However, questions remained: Which treatments are the right ones? How many are enough? And how much time is sufficient? In line with best practices recommended in countries permitting assisted dying (14), the Panel acknowledged that answering these questions in any given case would require carefully weighing the available options within a therapeutic dialogue between the clinician assessing eligibility and the individual seeking assisted death. However, to better define this process, further work would be needed to establish a clear approach for operationalizing the requirement of incurability.

Before proceeding, it is important to highlight that the Panel emphasized that the challenge of establishing the incurability of mental disorders was not in fact unique to such conditions. In fact, the approach it recommended was already being applied in assessments of nonpsychiatric chronic conditions permitted under existing legislation. To ensure a consistent approach across conditions, the Panel therefore recommended that:

The federal, provincial, and territorial governments should facilitate the collaboration of physician and nurse regulatory bodies in the development of Standards of Practice for physicians and nurse practitioners for the assessment of MAiD requests in situations that raise questions about incurability, irreversibility, capacity, suicidality, and the impact of structural vulnerabilities. (emphasis added) (2 p.12)

This recommendation indicated the Panel’s view that interpreting and operationalizing incurability could not be established simply by knowing a person’s diagnosis but required further knowledge about the nature of a requester’s particular condition and circumstances.

## 7 Model practice standard and Advice to the profession

Following this recommendation of the Federal Expert Panel for MAiD and Mental Illness, Health Canada established an independent MAiD Practice Standards Task Group. Because the Canadian Federal Government does not have jurisdiction over the regulation of clinical practice, the Group’s mandate was to develop a model regulatory standard that could then be adopted or adapted by the provincial and territorial medical and nursing regulatory bodies in updating their own MAiD standards (most regulators already had a standard that covered “Track 1” but had not yet been updated for “Track 2”). Importantly, regulatory standards are binding on members (in this case, physicians and nurse practitioners); therefore, a standard can establish an obligatory framework within which the assessment of incurability can occur.

Specifically, the Task Group paid attention to regulation with respect to the complexities introduced by cases where a person’s natural death was not reasonably foreseeable (including, but not limited to, cases where mental disorder is the person’s sole underlying medical condition). One of the Task Group’s aims was to operationalize the legal term “incurable” in a way that was applicable to the full range of conditions that could lead to a MAiD request.

The Task Group’s first step was to operationalize the Expert Panel’s recommendations. It then circulated a draft model practice standard and revised the substance in response to feedback from “all regulatory bodies for physicians and nurses in Canada, members of the MAiD clinical and research communities, health professional associations, and provincial/territorial ministries of health” (15). Following this process, Health Canada published two documents in March 2023: MAiD Practice Standards Task Group, Model Practice Standard for Medical Assistance in Dying (MAiD) (3) and Advice to the Profession: Medical Assistance in Dying (4).

The Model Practice Standard proposed the following interpretation of incurable:

9.5.2 “Incurable” means there are no reasonable treatments remaining where reasonable is determined by the clinician and person together exploring the recognized, available, and potentially effective treatments in light of the person’s overall state of health, beliefs, values, and goals of care. (3 p. 11)

The Advice to the Profession offered an additional explanation:

The incurability of the illness, disease, or disability does not require that a person has attempted every potential option for intervention irrespective of the potential harms, nor that a person must attempt interventions that exist somewhere in the world but are inaccessible to them. At the same time, a capable person cannot refuse all or most interventions and automatically render themselves incurable for the purposes of accessing MAiD. An assessor or provider cannot form an opinion about MAiD eligibility in the absence of evidence required to form that opinion, i.e., that there are no reasonable treatments remaining where reasonable is determined through a process of the clinician and patient together exploring the recognized, available, and potentially effective treatments in light of the patient’s overall state of health, beliefs, values, and goals of care. (4 p.4-5)

The Task Group’s proposed approach acknowledged that MAiD decision-making, *like all clinical decision-making*, must be considered within the context of a person’s health status, values, beliefs, and care goals. However, what happens if, after the joint exploratory process, the clinician and patient do not agree on whether there are “reasonable” treatments the patient has not tried? Further work on the operationalization of incurable remains necessary to address this question. Let us now turn to that project.

## 8 A proposal for further operationalizing the incurability requirement in MAiD eligibility assessments

Recall that the Model Practice Standard interpreted incurable as follows:

“‘Incurable’ means there are no reasonable treatments remaining where reasonable is determined by the clinician and person together exploring the recognized, available, and potentially effective treatments in light of the person’s overall state of health, beliefs, values, and goals of care.” (emphasis added) (3 p. 11)

To apply the legal term incurable to the full range of possible conditions that might lead to a MAiD request, it must be applicable even in circumstances where clinicians may not typically use the language of curability/incurability. These might include the following situations:

1. The person has refused a potentially effective and accessible treatment for their condition (e.g., vulvectomy for squamous cell carcinoma of the vulva).
2. There is confidence—but not certainty—about the prognosis and evolution of the person’s condition (e.g., multiple sclerosis).
3. The person’s condition is characterized by a constellation of symptoms, and/or its pathophysiology is poorly understood (e.g., multiple chemical sensitivities).

Other clinical terms have been developed to describe examples 2 and 3, such as treatment resistance (16), treatment refractoriness (17), or advanced or severe stage (18). These terms all indicate that usual therapeutic approaches have not achieved their targets.

Our proposed operationalization is derived from standard clinical reasoning across the full range of cases that may arise, integrating both idiographic and nomothetic approaches. This proposal also incorporates the evolving interpretation of the *Criminal Code* provision and respects the legal foundations upon which the operationalization project rests.

1. Clinicians should base their reasoning and judgment on information gathered from various sources, including the person’s medical records, input from clinicians directly involved in their care, collateral history from family and significant others (with the person’s consent), consultation with an expert in the condition causing the person’s suffering, specialist consultation, and relevant practice guidelines or scientific literature. If the person refuses to consent to obtaining the necessary information, the clinician must explain that the assessment cannot be completed. In such a case, it is not possible to determine whether the person’s condition is incurable.
2. Clinicians should assess the underlying nature of the person’s illness(es), disease(s), or disability/disabilities. Is the pathophysiology well-understood (e.g., glioblastoma [IDH-wildtype] or poorly understood (e.g., Adult-onset Still’s disease)? Does the underlying disease process correlate well (e.g., Crohn’s disease) or poorly with the symptomatic presentation (e.g., multiple sclerosis)? Is the condition defined entirely by its symptom profile (e.g., fibromyalgia)?
3. Clinicians should assess the treatments and interventions the person has undergone or has been recommended.
4. Clinicians should assess whether there are remaining treatment/intervention options that are recognized, available, and potentially effective for the condition. These include approaches that modify the disease process, if possible, and/or address its symptoms. If no such options exist, the clinician may determine that the person’s condition is incurable. If recognized, available, and potentially effective treatments do exist, the clinician should explore these options with the patient, considering the person’s overall health, beliefs, values, and goals of care. They should also discuss the reasons for the person’s refusal of treatment. Motivational interviewing techniques may help identify the underlying reasons for the refusal.
  - This may reveal that the proposed treatment is inappropriate for the patient, e.g., there are features specific to the person that might make the treatment unlikely to be effective. For example, while surgical treatment for colon cancer may require a permanent colostomy, the person may be unable to manage the demands of a colostomy bag. In such a case, the person’s condition can be considered incurable.
  - This may lead the patient to reconsider trying treatment. The initial refusal may have been based on misconceptions or fears about the treatment(s). In such cases, the clinician can provide further information and clarification to ensure the patient fully understands the treatment(s) being offered. If the refusal stems from fear, the clinician may help alleviate concerns or explore alternative approaches that the patient finds acceptable. For example, a person may initially reject a mastectomy for invasive breast cancer when breast-conserving therapy is not recommended. In this case, offering neoadjuvant therapy first may be an alternative the patient is willing to consider. If the person decides to proceed with treatment, their condition cannot yet be considered incurable.
  - The person may be unwilling to discuss available treatment options. In such circumstances, eligibility criteria—such as the requirement to provide informed consent—or procedural safeguards, such as the Track 2 requirement that the patient has “given serious consideration” to “reasonable and available means to relieve the person’s suffering”, may not be met. While this might impact eligibility, it does not determine the incurability of the condition and, therefore, falls outside the scope of this paper.
5. Where a person continues to refuse treatment after the exploration outlined above, the clinician should further assess what is known about the condition, specifically:
  - Where the person has a condition with potentially reversible pathophysiology and there is uncertainty about whether their refusal will persist over time—whether due to signs of indecision or because a significant period remains before irreversibility sets in—the condition cannot be considered incurable.
  - Where the person has a condition with a potentially reversible pathophysiology, even if a significant amount of time remains before irreversibility sets in, and there is confidence that their refusal will persist throughout that period, the condition can be considered incurable.
  - Where the pathophysiology is understood but the progression of symptoms is unpredictable, or where the pathophysiology is poorly understood and/or the condition is defined primarily by its symptom profile, the clinician should evaluate the remaining treatment options. Where a person has refused most available treatments, including some that are likely to alleviate symptoms, the condition cannot be considered incurable. However, where the person refuses the few remaining options, the condition can be considered incurable. In cases that fall between these extremes, the clinician encounters the inherent ambiguity in distinguishing between curable and incurable chronic conditions^19^. In such situations, they must rely on their best judgment, considering the totality of the clinical circumstances and the specifics of the case, to reach a decision.

## 9 Conclusion

The term incurable in MAiD policy and practice in Canada has had to evolve due to a changing legislative landscape. This, along with the absence of an authoritative definition of the term in law, has led to confusion and controversy regarding its interpretation and operationalization. In this paper, we have discussed the various attempts to interpret and operationalize the term and their various pitfalls. We have offered an approach to operationalizing the term in practice that is consistent with the current legal backdrop, the interpretations of the terminology used in the *Criminal Code*, and the clinical reality of the heterogeneous set of conditions that are (and in 2027 will be) potentially eligible for MAiD.

While our analysis is specific to Canada’s MAiD eligibility requirement of incurability, several permissive jurisdictions make use of the term incurable or related terms. Our proposal contributes to the international discussion and may be particularly useful to clinical organizations that also seek to develop guidance with respect to assessing this aspect of eligibility for assisted death and other jurisdictions that are considering the legalization of assisted dying.

## Footnotes

1
*Carter v. Canada (Attorney General)*, 2015 SCC 5 (*Carter*).

2 Bill C-14, *An Act to amend the Criminal Code and to make related amendments to other Acts (medical assistance in dying)* 1st Sess, 42nd Parl, 2016, (assented to 17 June 2016) (Bill C-14). Québec has its own additional MAiD legislation (which predates Bill C-14). Much of what follows in this paper is relevant to interpreting and operationalizing the Québec legislation; however, there are differences between the Québec and federal laws (e.g., the Québec legislation criterion is “serious and incurable illness”, while the federal legislation criterion is “serious and incurable illness, disease, or disability”. Mental illness is excluded under the federal legislation but mental disorder except for neurocognitive disorders is excluded under the Québec legislation. Given space constraints, an analysis of the interpretation and operationalization of the Québec provision lies outside the scope of this paper.

3 Bill C-14, supra note 2, s.3.

4
*Truchon c. Procureur general du Canada*, 2019 QCCS 3792 (*Truchon*)

5 Bill C-7, *An Act to amend the Criminal Code (medical assistance in dying)* 2^nd^ Sess. 43^rd^ Parl, 2021, c 2 (assented to 17 March 2021 s.1(1) (Bill C-7).

6 Bill C-7, supra note 5, s.1(2). “For the purposes of paragraph (2)(a), a mental illness is not considered to be an illness, disease or disability.”

7 Bill C-39, *An Act to amend an Act to amend the Criminal Code (medical assistance in dying)*, 1st Sess, 44th Parl, 2023 (assented to 9 March 2023) (Bill C-39) and Bill C-62, *An Act to amend An Act to amend the Criminal Code (medical assistance in dying)*, No. 2, 1st Sess, 44th Parl 2024 (assented to 29 February 2024) (Bill C-62).

8 Section 7 of the *Canadian Charter of Rights and Freedoms* establishes that “Everyone has the right to life, liberty and security of the person and the right not to be deprived thereof except in accordance with the principles of fundamental justice.” Section 15 establishes that “Every individual is equal before and under the law and has the right to the equal protection and equal benefit of the law without discrimination and, in particular, without discrimination based on race, national or ethnic origin, colour, religion, sex, age or mental or physical disability.” Section 1 establishes that limits on rights can sometimes be constitutional—i.e., if they are “prescribed by law” and “demonstrably justified in a free and democratic society”. *Canadian Charter of Rights and Freedoms, Part I of the Constitution Act*, 1982, being Schedule B to the *Canada Act* 1982 (UK), 1982, c 11.

9
*Carter supra* note 1, at para 127.

10 Chatham House Rule: “When a meeting, or part thereof, is held under the Chatham House Rule, participants are free to use the information received, but neither the identity nor the affiliation of the speaker(s), nor that of any other participant, may be revealed”. https://www.chathamhouse.org/about-us/chatham-house-rule

11
*Truchon*, supra note 4.

12 Between the *Truchon* decision and the passage of Bill C-7 (which amended the *Criminal Code* in response to *Truchon*), individuals could apply to the court for authorization to proceed with MAiD (http://eoldev.law.dal.ca/?page_id=2219). However, while these applicants did not have reasonably foreseeable natural deaths, incurability was not at issue. As as result, these cases did not further clarify the meaning of incurable.

13 Canada, Parliament, *Debates of the Senate*, 44th Parl, 1st Sess, Vol 153, No 179 (14 February 2024) at 1910

14
*Canada (Attorney General) v. EF*, 2016 ABCA 155 at paras 40 and 43-59.

15 Truchon, *supra* note 4 at 421 and 483-492.

16 “An adjudged case or decision of a court of justice, considered as furnishing and example or authority for an identical or similar case afterwards arising or a similar question of law.” The Law Dictionary https://thelawdictionary.org/precedent/#:~:text=Definition%20and%20Citations%3A,a%20similar%20question%20of%20law.

17 Some continued to assert that mental illnesses can never be said to be irremediable as clinicians can never be certain that the person will continue to have the symptoms of the condition. Given the position taken by Parliament and the Government set out above, these assertions will be considered moot and will not be addressed.

18 The Federal Expert Panel retained the expression “mental illness” in its name because this was used in its official mandate from the government. However, it noted that the term mental illness does not have a standard definition and therefore used the expression “mental disorder” throughout its report because this is the term used in both major diagnostic classification schemes relied upon in Canadian psychiatric practice: the American Psychiatric Association’s Diagnostic and Statistical Manual of Mental Disorders (DSM) and the World Health Organization’s International Classification of Diseases (ICD). (2, p6) Mental disorder was also the recommended term in the comprehensive review of the knowledge available on the topic of MAiD for mental illness required by the 2016 MAiD legislation. (13)

19 This is an instance of the classic sorites paradox. Imagine you have a heap of sand. If you remove one grain, you still have a heap. Remove a few more, you still have a heap. However, if you continue removing grains, you will eventually be left with only a few, at which point it is no longer a heap. The question is: when does the transition from heap to nonheap occur? It is impossible to provide the exact number of grains at which this change takes place, even if there is agreement on what constitutes a heap and what does not. Similarly, while clinicians may agree on whether a condition is incurable or curable, it may not be possible to specify after which failed treatment trial this transition occurs.
